# Psychosocial impact of false-positive surveillance for hepatocellular carcinoma: a qualitative study

**DOI:** 10.1007/s00520-026-10559-4

**Published:** 2026-03-24

**Authors:** Samuel Hui, Suong Le, Anouk Dev, Sally Bell

**Affiliations:** 1 School of Clinical Sciences at Monash Health, Monash University, Melbourne, Australia; 2 Department of Gastroenterology and Hepatology, Monash Health, Melbourne, Australia

**Keywords:** Hepatocellular carcinoma, Cancer screening, Psychosocial harm, False-positive diagnosis, Qualitative study

## Abstract

**Purpose:**

Biannual ultrasound-based hepatocellular carcinoma (HCC) surveillance is standard practice for individuals with cirrhosis and subgroups with non-cirrhotic hepatitis B. While this practice improves cancer-related survival, the detection of false-positive results can lead to additional testing which poses both physical and psychosocial risks. Our study explored the psychosocial consequences of false-positive ultrasound results in participants undergoing HCC surveillance.

**Methods:**

We performed a qualitative study with semi-structured interviews to understand the psychosocial impact of participants who received a false-positive ultrasound result during HCC surveillance until no new themes emerged. Key themes were analysed using the Framework Approach.

**Results:**

Ten participants with cirrhosis or non-cirrhotic hepatitis B were recruited. Most had a poor understanding of the role of ultrasound in HCC surveillance. Some participants experienced significant anxiety with existential concerns impacting family and work life. Others remained uninformed about the risk for HCC and did not experience significant psychosocial impacts. The lack of understanding about the role of surveillance and the potential for false positives contributed to unpredictable psychosocial consequences. Despite experiencing surveillance-related harm, all participants wished to continue regular surveillance.

**Conclusion:**

Our findings highlight a need for improved health literacy about the purpose and potential harms of HCC surveillance. Optimising surveillance protocols to minimise false-positive findings may also further alleviate physical and psychosocial harm.

## Background

Hepatocellular carcinoma (HCC) is a leading cause of cancer-related death worldwide [1]. It is an aggressive malignancy that most often occurs in people with underlying chronic liver disease and particularly cirrhosis.

Biannual ultrasound-based HCC surveillance, with optional inclusion of the serum alpha-fetoprotein, is recommended by all major hepatology societies for people with cirrhosis and certain subgroups with non-cirrhotic hepatitis B [2–4]. Surveillance results in early detection of HCC, an increased possibility of delivering curative treatment and improved survival [5].

While the use of ultrasound rarely leads to direct physical harm, its imperfect specificity may lead to false-positive results, which could prompt further diagnostic testing [6]. These subsequent tests carry the risk of physical harm, such as radiation exposure associated with computed tomography (CT) scans, contrast-related complications or invasive procedures such as biopsy or unnecessary surgery. Observational studies have estimated that 8–28% undergoing HCC surveillance may experience these risks [7–9].

The need for follow-up tests due to false-positive ultrasound results can lead to psychosocial harm. This may manifest as anxiety, disruptions to work and personal commitments, and diminished trust in cancer screening. Such harm from false-positive results has been described in other surveillance programs, including mammography for breast cancer screening and faecal occult blood testing for bowel cancer screening [10, 11].

Narasimman et al.’s study is the only study to have evaluated non-physical harms associated with HCC surveillance in people with cirrhosis. The authors incorporated a survey-based evaluation as well as semi-structured interviews across several centres in the USA and focused on the psychological harms of HCC surveillance [12]. Overall, they found that psychological harm occurred for both true positive and false positive participants in HCC surveillance, although this harm appeared milder than in breast or prostate cancer screening programs.

Building on this existing work, our study extends the evaluation of HCC surveillance to a broader psychosocial context for participants who experience a false-positive finding. We aimed to track the participants’ journey from initial detection of a suspicious lesion, through to the experience of undergoing additional investigations and finally their perspective of HCC surveillance. We were particularly interested in evaluating a participant’s understanding of HCC surveillance, and how this impacted the psychosocial consequences when a false-positive lesion was detected.

## Methods

### Study population and recruitment

Our study was conducted in Melbourne, Australia, with recruitment from participants who attend liver clinics at our institution. Potential participants were identified through a combination of purposeful sampling from our institution’s HCC surveillance database as well as opportunistic sampling by clinicians at the liver clinics. Participants were eligible for inclusion if they had a history within the past 5 years of a benign liver lesion identified through ultrasound-based HCC surveillance for cirrhosis or non-cirrhotic hepatitis B that necessitated further investigation. Participants were excluded if the false-positive liver lesion was initially detected on non-surveillance-related scans. We conducted interviews until thematic saturation was reached.

### Semi-structured interviews

We used semi-structured one-on-one telephone interviews to explore participants’ experience of a diagnosis of a false-positive liver lesion during HCC surveillance. The interviews were conducted by the principal investigator (SH) between September 2022 and February 2025 and lasted 30–40 min. The principal investigator is a specialist hepatologist and has undergone additional training in qualitative research methods. The interviewer did not have an ongoing clinical relationship with participants and participants were assured that their responses would not affect their clinical care.

We used four key questions as a framework for the interview, and these were agreed upon by all the authors based on a review of the literature and the authors’ clinical experience (Table 1). Deeper responses to the questions were elicited through probing techniques. All interviews were audio recorded and transcribed verbatim.

**Table 1:** Key questions used in semi-structured interview

What is your understanding of liver cancer surveillance?
When you found out you had a mass in your liver, how did this impact you?
How did you find the experience of going through additional tests? (biopsy, additional scans etc.)
How will your prior experiences with liver cancer screening affect your future participation in surveillance?

### Data analysis

Interview transcripts were coded for key themes using the Framework Approach [13]. The analysis was conducted by SH and began with a comprehensive review of the data by repeated readings of the transcripts. Both a priori and emergent codes were applied during this analysis. A priori codes were derived from the literature and the study’s four key questions, while emergent codes reflected new insights that emerged directly from the participants’ interviews.

The coded data was then organised into a thematic framework which was further refined through iterative analysis. This iterative process involved revisiting and refining the data as new themes emerged. The framework approach was selected as it allows structured analysis of predefined clinical questions while remaining open to emergent participant experiences.

### Ethics

This study was approved as a low-risk research activity by the Monash Health Human Research Ethics Committee, in accordance with the Declaration of Helsinki. Informed consent was obtained from participants and all transcripts were de-identified. Participants were not offered any reimbursement.

## Results

Ten participants were interviewed with a median age of 51 years (interquartile range 44–58) (Table 2). Four participants (40%) were male. The most common aetiology of liver disease was hepatitis B (60%). One participant was recorded for each of the following aetiologies: metabolic dysfunction-associated steatotic liver disease (MASLD), metabolic dysfunction-associated alcohol-related liver disease (MetALD), hepatitis C, and alcohol-related liver disease (ALD). Fifty percent of the participants were cirrhotic. There was significant cultural diversity in our cohort, reflective of the demographics who experience liver disease and undergo HCC surveillance in our region.

**Table 2:** Baseline characteristics

Number of participants	10
Age at interview, years, median (interquartile range)	53 (43–78)
Sex	
- Male	4 (40%)
- Female	6 (60%
Ethnic background	
- East Asian	4 (40%)
- South Asian	1 (10%)
- Pacific Islander	1 (10%)
- Caucasian	4 (40%)
Cirrhosis	5 (50%)
Primary aetiology of liver disease	
- Hepatitis B	6 (60%)
- Hepatitis C	1 (10%)
- Metabolic dysfunction associated steatotic liver disease (MASLD)	1 (10%)
- Alcohol-related liver disease (ALD)	1 (10%)
- Metabolic dysfunction associated alcohol-related liver disease (MetALD)	1 (10%)

The key themes are presented in chronological order based on the participant experience during the workup for a positive ultrasound result.

### Variable understanding of the role of regular ultrasound surveillance and individual HCC risk

Most participants did not understand why ultrasound surveillance was being undertaken. Only one participant could clearly articulate that the purpose of ultrasound examinations was for the detection of liver cancer, while the remaining participants reported the use of regular ultrasound surveillance was for the assessment of their overall liver health. Additionally, most participants did not recall specific discussions with their healthcare providers about the role of regular ultrasound surveillance, instead assuming it was for general monitoring. Some of the assumptions about regular ultrasound surveillance included:

“Monitoring for fatty liver syndrome or whatever you used to call it.” – 65M, MetALD

“I have a history of alcohol which damages your liver, it’s like just checking that it’s not getting any worse or any better.” – 43 F, ALD

“Not really sure. I think they’re looking to see if the surface of the liver is getting bigger”. – 61 F, HCV

When probed, several participants, however, could identify that they were at increased risk of developing HCC due to their chronic liver disease, though it was not something they frequently considered. As one participant reported:

“I guess so but you’re kind of in a bit of denial I guess” 61 F, HCV

Some participants felt they had no significant risk of liver cancer due to reassuring results. One participant felt that their regular attendance at ultrasound would eliminate the risk of liver cancer, rather than serving as a tool for early detection.

“I don’t think so because like every half or one year I need to go and do blood tests and stuff (ultrasound)” 45 F, HBV

### Anxiety associated with initial detection of a liver mass

Six participants reported anxiety upon the detection of a liver mass, with some reflecting on existential thoughts. These thoughts were often centred around the impact of a potential cancer diagnosis on their family. Some of these sentiments included the following:

“I felt very depressed, like stressful anxiety.” – 52 F, HBV

“At the time, definitely, there was concern. I knew that the spot wasn’t something good.” – 78M, MASLD

“I’m a full time carer for someone who’s on the spectrum.” – 61 F, HCV

“If I live or if I die, it doesn’t really matter that much to me. I do it for others really.” – 65M, MetALD

Some participants chose to share the news of the lesion detected on their ultrasound with family members, while others opted to keep it to themselves, concerned about the impact it might have on their loved ones. Those who chose to disclose the information reported that their family members often became more distressed than the actual participant.

One participant highlighted how the manner of communication from the provider may impact the anxiety experienced with the detection of a liver mass.

“I got a call from the specialist, they were absolutely hysterical which made me hysterical.” – 61 F, HCV

On the other hand, four participants reported no significant anxiety as they were asymptomatic and lacked understanding or awareness of the potential concern for HCC. These participants also did not recall their healthcare provider emphasising the potential for an HCC diagnosis.

“I didn’t feel unwell and there was sort of…nothing that indicated anything sort of sinister.” – 48 F, HBV

One participant, who spoke English as a second language, reemphasised the importance of the language used by healthcare providers.

“I didn’t really understand what that word means”, the participant said, referring to when a liver ‘lesion’ was detected. – 45 F, HBV

### Variable impact of additional tests

The physical and psychosocial impact of additional tests varied depending on the type of workup and the individual circumstances of the participants.

One participant underwent surgery for a lesion that was ultimately benign. This had profound psychological and financial consequences on the participant’s family due to hospitalisation, prolonged work absenteeism, and their partner requiring carers leave.

Several participants reported discomfort and claustrophobia during magnetic resonance imaging (MRI) scans. One participant also expressed anxiety about radiation exposure related to computed tomography (CT) scans. No participant identified the time away from work or other activities for additional scans to be a problem, as they generally viewed these tests as a one-time occurrence. As one participant put it:

“I don’t mind as it’s like my insurance to make sure my body is ok” – 58M, HBV

### Strong desire to continue long term surveillance

Despite the frequently reported anxiety associated with false-positive lesions and the subsequent workup, all participants expressed a strong desire to continue long-term ultrasound-based HCC surveillance. This occurred even amongst participants who experienced significant distress, discomfort, or inconvenience generated by the additional workup following a false-positive result.

In many cases, the experience of a perceived threat of cancer became a motivating factor for participants to take HCC surveillance seriously. This also pertained to participants who could not clearly link the role of regular ultrasounds to HCC surveillance. One participant highlighted this motivation in describing:

“I’ll keep going. If the lesions do eventually appear again, I want them to get caught out and taken off.” – 61 F, HCV

Many participants had undergone ultrasound-based HCC surveillance for many years and had accepted it as an essential part of their routine healthcare. One participant explained:

“It’s all just sort of part of my life now”. – 43 F, ALD

Despite experiencing the limitations of HCC surveillance, most participants expressed strong trust in the healthcare they were receiving, which appeared to be a key motivator for their desire to continue surveillance. Only one participant reported conflicting thoughts about the validity and accuracy of HCC surveillance scans when they pondered:

“And then you’d kind of wonder well is there something then they’ve missed? You know, if they’ve made this mistake, could they make another mistake?” 61 F, HCV

## Discussion

This is the first qualitative study to specifically investigate the psychosocial impact of HCC surveillance harm. This study gap needed to be addressed in order to improve the implementation, effectiveness and acceptability of HCC surveillance programs and its impact on long-term surveillance.

Firstly, our study highlights the crucial need for ongoing patient education for individuals enrolled in HCC surveillance. This contributes to better patient engagement in surveillance but may also alleviate anxiety when positive findings are detected. Most participants had a poor understanding of HCC surveillance and could not link their regular ultrasound examinations to the explicit purpose of HCC surveillance. This may stem from the fact that HCC surveillance forms only one aspect of care that participants receive at liver clinics, making the exact purpose of ultrasound less obvious when compared to screening programs in healthy populations, such as those for breast and bowel cancer. Additionally, healthcare providers may provide insufficient education on this topic, potentially only at the start of a participant’s engagement with the liver clinic, rather than providing ongoing clarification. Nevertheless, most participants were able to identify their increased risk of HCC due to their known history of hepatitis B and cirrhosis.

This gap in participants’ health literacy and knowledge may contribute to unpredictable psychosocial consequences when a lesion is detected. Some participants remained oblivious of the potential concern for HCC and did not therefore report heightened anxiety. This may particularly occur when healthcare providers use medical jargon that minimises the perceived risk. Conversely, some participants experienced extreme anxiety and existential thoughts. This anxiety permeated all aspects of some participants’ lives, contributing to time away from work and family members also experiencing distress. This would be consistent with the findings by Narasimman et al. who reported a high frequency of participants engaging religious and external support systems for both true-positive and false-positive ultrasound findings [12]. In our study, we hypothesise that the lack of baseline understanding regarding the role of regular ultrasound-based surveillance particularly contributes to the unexpected and distressing experience for some participants when informed of a positive ultrasound result.

The experience of undergoing additional workup was highly individualised, based on the participants’ experience and tolerance of additional investigations, as well as the nature of the investigations themselves. This would be consistent with the variable experience reported in the literature for anxiety related to medical imaging procedures, such as MRI related claustrophobia and patient perceptions of CT contrast related risk [14, 15]. No participants were concerned about the inconvenience generated by additional investigations, due to the perception this was a one-time occurrence.

Although most participants lacked a firm understanding of the role of regular ultrasound-based surveillance, the experience of a false-positive finding was a motivating factor and led all participants to want to continue their engagement with surveillance. This is noteworthy given this cohort experienced negative consequences of imperfect cancer screening.

Psychosocial harm from false-positive results has been described across multiple cancer screening programs; however, the context in which screening occurs may substantially shape how this is experienced. In the study by Narasimman et al., the authors reported milder psychological harm for HCC surveillance participants when compared to other cancer screening programs [12]. They hypothesised this was related to a degree of psychological blunting and fatalism for people with cirrhosis when approaching their HCC risk, in contrast to healthy participants in other screening programs. In our analysis, we similarly observed a sense of resignation and acceptance amongst several of the participants who had more advanced liver disease and had prior lived experience of liver disease related complications. Conversely, the greatest psychological distress and anxiety were experienced by individuals with non-cirrhotic hepatitis B, who are unlikely to have previously encountered other complications of liver disease.

### Strengths and limitations

Our study provided an in-depth exploration of the psychosocial impact of participants who receive a false-positive result during HCC surveillance. The qualitative design with the use of in-depth semi-structured interviews offers rich data that would not be possible to capture with a quantitative or survey-based design. We additionally sampled from a diverse cohort not only in respect to cultural diversity, but also with regard to HCC risk and liver disease aetiology. An additional strength was the longitudinal nature of our interviews, which allowed for the exploration of how participants’ perspectives evolved following the detection of a false-positive finding. This helped capture both the short and long-term psychosocial impacts of false-positive results.

We also highlighted the need to improve the surveillance process itself, so that the risk of false-positive findings is minimised. Risk-based stratification in particular has been proposed as a strategy to optimise sensitivity for higher-risk patients while also minimising harms related to false-positive findings for lower-risk patients, such as those with non-cirrhotic hepatitis B [16]. In a potential risk-stratified protocol, these lower-risk patients may be screened with novel biomarker panels such as the GAAD and GALAD, which have a high specificity for early-stage HCC detection in recently reported validation studies [17, 18].

The small sample size of our study is a limitation and the experiences reported by our cohort do not necessarily represent the full spectrum of individuals undergoing HCC surveillance. Despite the small sample size, thematic saturation was reached through the in-depth interviews and the main research objectives were fulfilled.

Our study is also vulnerable to selection bias as those who participated may be more engaged and interested in their liver disease and HCC surveillance. Despite this, our study highlighted significant limitations in participants’ health literacy and knowledge with regards to HCC surveillance.

We used telephone rather than in-person interviews, which limited access to non-verbal cues. However, evidence comparing interview modes suggests the main differences relate to interactional features rather than interview content [19, 20]. Given that our study focused on participant experience, telephone interviews were appropriate for the study aim while also increasing accessibility for participants.

Finally, in our study, participants were not involved in the co-design of the interview guide. The interview questions were developed by the research team based on existing literature and clinical experience. While patient co-design may enhance relevance and depth of analysis, the semi-structured format allowed participants to guide the discussion toward issues most salient to them, and thematic saturation was achieved. Incorporating patient co-design in future studies may further strengthen the relevance and acceptability of qualitative research exploring harms of HCC surveillance.

## Conclusion

Our study has shown that some people experience significant short and long-term psychosocial consequences of false-positive findings from HCC surveillance. We also found notable shortcomings in participants’ health literacy and understanding of the role of ultrasound-based HCC surveillance. This lack of knowledge results in unpredictable psychosocial consequences when false-positive findings arise. Future studies should focus on further evaluating the healthcare literacy of HCC surveillance participants and exploring the psychosocial impact of surveillance in different healthcare settings, including primary care and regional or remote areas.

## Data Availability

No datasets were generated or analysed during the current study.
